# Sengstaken Tube Removal under Direct Hemodynamic Monitoring after Post Transplantation Venous Occlusion

**DOI:** 10.1155/2019/6146125

**Published:** 2019-08-29

**Authors:** A. L. Colón, L. Rodríguez-Bachiller, E. Velasco, B. Díaz-Zorita, D. Rincón, J. A. López-Baena, J. Ferreiroa

**Affiliations:** ^1^Liver Transplant Unit, Department of General Surgery, Hospital General Universitario Gregorio Marañón, Madrid, Spain; ^2^Liver Transplant Unit, Department of Hepatology, Hospital General Universitario Gregorio Marañón, Madrid, Spain

## Abstract

The surgical procedure for orthotopic liver transplantation (OLT) is well standardized, and most groups use the retrohepatic caval preservation or piggyback technique to improve hemodynamic tolerance. However, when a discrepancy between the site in the right upper quadrant of the liver recipient and a small graft is present, this technique can provoke a rotation on the axis of the vena cava and cause an occlusion of the suprahepatic vein drainage. This problem can be detected intraoperatively, and several methods have been described to resolve it by placing different devices to correct the position. Early withdrawal may cause the development of clinical hepatic congestion with ascites unresponsive to medical treatment. We present three cases of OLT who developed obstruction of the venous drainage solved intraoperatively with the placement of a Sengstaken-Blakemore tube. As a novelty, prior to the withdrawal of the device, a transjugular hemodynamic study was performed to ensure the correct position of the liver with adequate venous drainage.

## 1. Introduction

Orthotopic liver transplantation (OLT) represents the best definitive treatment for patients with cirrhosis and some variants of tumors such as hepatocellular carcinoma. The surgical procedure is well standardized, and most groups have adopted the piggyback technique which consists in suturing the proximal vena cava stump of the graft to the common suprahepatic vein ostium in the recipient [1, 2]. With this maneuver, hemodynamic tolerance is improved, as opposed to the classical technique (which requires retrohepatic vena cava resection), avoiding complete venous clamping and the eventual need of a veno-venous bypass.

However, if there is a discrepancy between the size of a small graft and a liver recipient with an oversized site in the right upper quadrant (e.g., patients with ascites), using the piggyback technique can cause a liver malposition due to rotation on the axis of the vena cava which produces an occlusion of the suprahepatic veins resulting in an acute Budd-Chiari syndrome, with ascites, impaired liver function, and risk of graft loss [3].

Usually, this problem is detected intraoperatively, and several methods have been described in the literature to resolve this malposition/rotation by placing different devices such as Foley probes [4], breast prosthesis [5], or a Sengstaken-Blakemore tube [6, 7].

Removal of these devices such as the Sengstaken-Blakemore tube or the Foley catheter is usually performed after a few days without any control, assuming that the liver graft has already formed adhesions to the parietal peritoneum and is permanently well positioned.

Early withdrawal may cause the development of clinical hepatic congestion with ascites unresponsive to medical treatment, which may sometimes require the placement of prostheses into the vena cava to rectify the anastomosis increasing the risk of further complications [8].

The transjugular hemodynamic study is a safe method to quantify portal hypertension. It can be performed in an ambulatory setting with local anesthesia and with a very low complication rate. The procedure has been previously described by our group [9, 10]. A vascular introducer sheath (MEDIKIT Co. Ltd., Tokyo, Japan) was placed into the right internal jugular vein. Under fluoroscopic control, a 7F balloon catheter (Cordis SA, Miami, FL, USA) was guided into the right hepatic vein and inferior vena cava for the measurement of hepatic venous pressure (HVP) and vena cava pressure (VCP). The transanastomotic hepatocaval gradient (THCG) was calculated as HVP minus VCP. In a normal situation, we considered a THCG between 0 and 3 mmHg as normal.

We present three cases of OLT who developed obstruction of the venous drainage of the liver caused by excessive rotation and were solved intraoperatively with the placement of a Sengstaken-Blakemore tube. As a novelty, prior to withdrawing the device, a transjugular hemodynamic study was performed to verify the absence of a transanastomotic gradient and the correct venous drainage of the liver. The report is according to the CARE guidelines [11].

## 2. Patients

### 2.1. Case 1

The first case is a 51-year-old male with HCV+genotype 3a chronic liver disease. OLT is indicated because of the presence of a hepatocellular carcinoma (HCC) complying with the Milan criteria. The physical measures of the patient were as follows: height, 176 cm; weight, 90 kg; abdominal perimeter, 109 cm; and thoracic perimeter, 111 cm. A first OLT was performed with a graft from an 81-year-old donor. In the first 48 hours, the patient developed severe primary malfunction, for which a second OLT was indicated. He received a graft from a second donor with the following measures: height, 160 cm; weight, 68 kg; abdominal perimeter, 93 cm; and thoracic perimeter, 95 cm. The recipient/donor weight ratio was 1.3. The surgery was performed following our habitual technique with vena cava preservation (piggyback) with an anastomosis between a common stump from the recipient suprahepatic veins and the donor's cava after completely resecting the cuff of the previous graft's vena cava.

**Figure 1:**
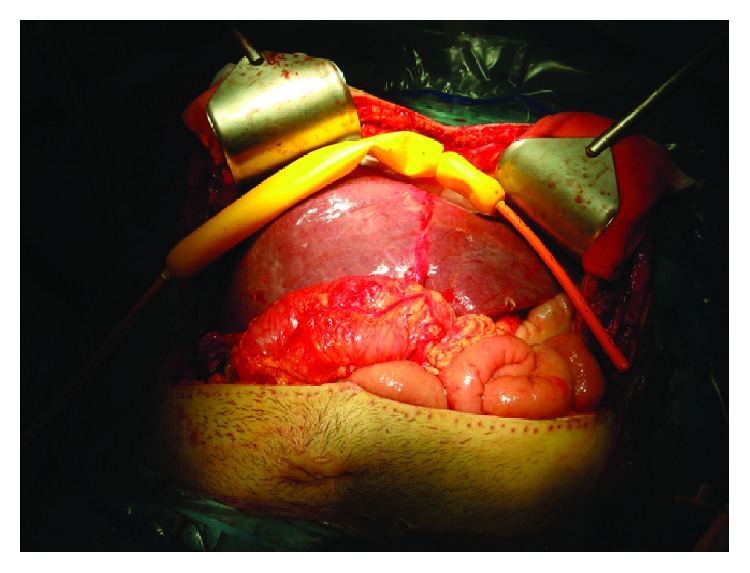
A Sengstaken-Blakemore tube placed under the liver in the right hypochondrium with the gastric and esophageal balloons filled.

In the course of the intervention, a rotation of the liver was observed due to a discrepancy between the graft's size and the space in the hepatic fossa that impaired the venous drainage causing hepatic congestion. To solve this problem, a Sengstaken-Blakemore tube was placed under the liver in the right hypochondrium with the gastric and esophageal balloons filled, thus correcting its rotation and achieving an immediate improvement in the venous drainage (Figure 1).

In the 7th postoperative day, a liver hemodynamic study was performed through the jugular vein. The THCG was measured with the balloons full and empty, observing that it did not increase after deflating them, so subsequent removal of the catheter was deemed safe. The patient was discharged 4 weeks after the surgery with optimal liver function, without ascites or other associated complications.

### 2.2. Case 2

The second case is a 39-year-old patient with familial amyloid polyneuropathy with associated gastrointestinal involvement. OLT was performed with a “domino” strategy (the explanted liver was used as a graft for a compatible liver recipient with decompensated cirrhosis). The physical parameters were as follows: height, 1.90 cm; weight, 90 kg; abdominal perimeter, 100 cm; and thoracic perimeter, 107 cm. An OLT was performed using a graft from a donor with an abdominal perimeter of 99 cm and thoracic perimeter of 95, with a weight of 68 kg, and a size of 163 cm. The recipient/donor weight ratio was 1.3.

A piggyback technique was performed as described. After reperfusion of the graft, hepatic congestion was observed due to poor venous drainage in relation to excessive rotation of the anastomosis of the suprahepatic vena cava. This was corrected by placing a Sengstaken-Blakemore tube in the right hypochondrium with both balloons inflated raising the liver, thus modifying the position of the liver and achieving adequate venous drainage. The postoperative course was uneventful. In the 7^th^ postoperative day, a direct hepatic hemodynamic study was performed through the jugular vein checking the absence of a THCG after deflating the Sengstaken-Blakemore tube's balloons proceeding to its withdrawal. The patient was discharged with a correct hepatic function, without ascites or other associated complications.

### 2.3. Case 3

The third case is a 51-year-old male patient with a 3 cm HCC. In the period on the waiting list, 2 treatments were performed with transarterial chemoembolization (TACE). Arterial spasm during one of them occurred as a complication. The physical parameters were as follows: size, 179 cm; weight, 82 kg; abdominal perimeter, 102 cm; and thoracic perimeter, 106 cm.

OLT was performed with the piggyback technique. He required acute retransplantation in the first 48 hours due to arterial thrombosis.

The second graft came from a donor with the following parameters: size, 165 cm; weight, 63 kg; abdominal perimeter, 95 cm; and thoracic perimeter, 90 cm. The recipient/donor weight ratio was 1.3. The second procedure was performed following the same piggyback technique, and the arterial intake necessitated the interposition of an aortohepatic graft. The disproportion of size conditioned a rotation on the axis of the caval anastomosis causing venous congestion. A Sengstaken-Blakemore tube with both balloons inflated was used to correct the position. A first liver hemodynamic measure was carried out in the 6th postoperative day, which showed an augmented THCG (6 mmHg) after deflating the gastric balloon of the Sengstaken-Blakemore tube. The balloon was again inflated, correcting the hepatic venous hypertension, so it was maintained in place for 72 hours more. A second measure was then performed showing a favorable result after emptying the balloon, so the tube was withdrawn without incident. The patient was discharged two days later in good clinical condition.

## 3. Discussion

**Figure 2:**
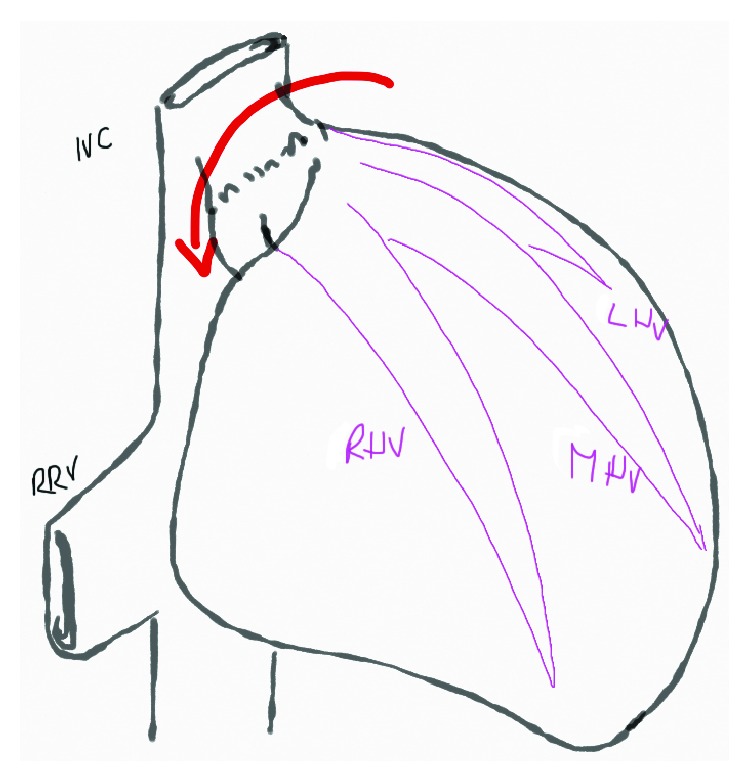
Malrotation is associated with the piggyback technique usually when there is a discrepancy between the graft size and the recipient volume. IVC: inferior vena cava; RRV: right renal vein; LHV: left hepatic vein; MHV: middle hepatic vein; RHV: right hepatic vein.

The surgical procedure for liver transplantation has become highly standardized, and most groups perform vena cava preservation with the piggyback technique, using an end-to-side anastomosis from the donor's caval stump to a cuff tailored with the recipient's suprahepatic veins. One of the reported complications is acute obstruction of the hepatic venous drainage, which is usually associated with technical problems related to anastomotic stenosis or liver malrotation. This event is more frequent when using the piggyback technique (3-4%) [12] than with the classic cavo-caval anastomosis technique (2%) [13] causing ascites refractory to medical treatment and impaired liver function augmenting the postoperative mortality (Figure 2).

Two of the three cases presented here occurred after acute retransplantation. In these occasions, a longer venous cuff is usually obtained by leaving in place the previous anastomosis and using the failed graft's proximal cava, thus facilitating the second anastomosis and reducing the necessity of partial clamping of the recipient's cava [14]. However, this may facilitate a posterior graft rotation and venous occlusion.

By the same causal mechanism, this regional Budd-Chiari syndrome can occur after a right hepatectomy due to a rotation of the left hemiliver, impairing the venous drainage through the trunk of the left suprahepatic vein [15].

In the field of transplantation, this complication occurs more frequently in pediatric and living donor settings. It can also happen when using cadaveric adult donors if there is a discrepancy between the graft size and the recipient volume and the piggyback technique is used, and the probability is even higher in retransplantation where the urgency of the case does not allow to adapt the perimeters and sizes between the donor and the recipient. A recipient/donor matching is usually attempted in our institution according to the weight and the thoracic and abdominal perimeters. Unfortunately, sometimes there is no choice but to use grafts from smaller donors in larger recipients where rotating problems such as those described by the size discrepancy may occur.

Diagnosis is usually done intraoperatively, becoming evident after reperfusion when the graft acquires a congestive, bloated aspect due to poor venous drainage, and is corrected by rectifying its position in the right hypochondrium, elevating it to avoid rotation of the suprahepatic veins.

Various solutions have been reported to avoid this rotation and maintain a correct position of the liver, such as the placement of a breast prosthesis behind the right liver. This procedure has the disadvantage that the prosthesis cannot be removed without a new surgical procedure [5]. Other methods described are the placement of a Sengstaken-Blakemore tube [6, 7] or a subhepatic Foley catheter [4].

In the reported cases, the removal of these devices is performed “blindly” assuming that the adhesions of the liver to the peritoneum and its adaptation to the new space can maintain the correct position. However, if this is not achieved, malfunctioning of the graft may occur after removing the device and the patient develops a Budd-Chiari-like syndrome, with ascites refractory to conventional medical treatment and impaired liver function.

In order to avoid this, we propose to do a direct hemodynamic study via right internal jugular vein to measure THCG before and after deflating the balloon, so that we can be sure that once the device is removed, the liver will drain correctly. Maybe with a Doppler study, we can obtain similar information on the inversion of the flow at the level of the suprahepatic veins, but the acute local inflammatory changes together with the interference produced by the Sengstaken-Blakemore's tube makes the assessment more difficult using an ultrasound approach. We preferred to make a hemodynamic study, and despite being an invasive technique, we considered that this approach is a safe procedure and provides us relevant information before removal of the device in real time, monitoring the correct drainage through the suprahepatic veins.

The optimal moment for the tube removal is in the early postoperative course but no sooner than 5 days after the surgery, to permit the formation of natural adhesions that will keep the organ in place. In our institution, the study is performed by the hepatology team with accumulated extensive clinical experience. This study can be performed easily with a few complications.

In conclusion, the placement of a removable device such as a Sengstaken-Blakemore balloon intraoperatively helps to correct the position of the liver avoiding its malrotation and obstruction of the venous drainage. Performing a hemodynamic study before its withdrawal allows to objectively verify adequate venous drainage.
